# A systematic review of the effects of neuromodulation on eating behaviour: implications for brain directed treatments in eating disorders

**DOI:** 10.1186/2050-2974-1-S1-O70

**Published:** 2013-11-14

**Authors:** Jessica McClelland, Ulrike Schmidt, Iain Campbell, Natalie Bozhilova

**Affiliations:** 1 Institute of Psychiatry, Kings College London, UK

## 

Eating disorders (ED) are chronic, deadly illnesses and especially for adults with anorexia nervosa, existing treatments have limited proven efficacy. Growing knowledge about the neural underpinnings to ED provides an avenue for more targeted, brain directed interventions. Brain stimulation techniques, such as Transcranial Magnetic Stimulation (TMS), transcranial Direct Current Stimulation (tDCS), Vagus Nerve Stimulation (VNS) and Deep Brain Stimulation (DBS) have the ability to directly alter neural activity within the brain. Such methods are being used extensively within both research and clinical settings to treat movement disorders such as Parkinson's and a range of psychiatric illnesses including depression. Findings within such disorders, relevant animal models and more recent research within ED populations have led to a strong rationale for the use of brain stimulation in ED. This paper systematically reviews this literature, identifying deficits in the current knowledge in an attempt to help guide future research and clinical practice within the imminent field of brain directed interventions for ED.

This abstract was presented in the **Understanding and Treating Eating Pathology** stream of the 2013 ANZAED Conference.

